# Interdisziplinäres Autorenteam Witten (Hrsg.): Heal Your Hospital. Studierende für neue Wege der Gesundheitsversorgung

**DOI:** 10.3205/zma001150

**Published:** 2018-02-15

**Authors:** Friederike Störkel

**Affiliations:** 1 FH Münster, Fachbereich Gesundheit, Münster, Germany

## Recension

This book is remarkable: students of medicine, psychology, economic sciences, philosophy and cultural sciences come together as part of their compulsory fundamental studies to tackle the subject "Heal your Hospital". The approach chosen incorporates three key aspects of an academic education: (specialist) knowledge, personal development, and preparation for the labour market [1]. Nine students remain faithful to their topic, giving voice to their "power for initiative and creative drive" as future "healthcare professionals" [2] by addressing their professional role, their professional self-awareness, their patients, structures of care, and the health system. They scrutinise how things have been done up until now, encourage the reader to reflect and think about their own positioning, and present ideas for how to rethink things and think afresh, which they summarise in eleven theses at the end of the book, resisting the temptation to present hasty solutions. This bears witness to specialist expertise and professional humility.

Over the course of nine chapters, the range of issues covered stretches from the individual in personalised medicine right up to a "Foray through the Health System" (Chapter 7). Light is cast on shared decision-making, health literacy, the working conditions of healthcare professionals, the difficulties of intra- and inter-professional communication, the mastery of interfaces, and the situation that hospitals find themselves in, in that area of tension between being a profit-oriented organisation and the opportunity for a common cultural and organisational development process. The authors also address the strengths and weaknesses of the principle of solidarity in the health care system, the possibilities for prevention and integrated care, and the question about the quality of the services provided. The sources used to back up argumentation testify to an intense debate on the topic in question. This is clear, for example, in the chapter on "Trust through Interaction", where, among others, statements by Badura and Feuerstein from 1996 are revisited, which even "back then" made it clear that interfaces within the scope of patient care have a particularly high need for social design that can't be met by purely technical solutions. Welcome to the here and now! The fact that this chapter includes an examination of the subject of communication in organisations based on Luhmann's systems theory underlines just how extensively this topic was reflected upon. Overall, the author team present themselves, in the truest sense, as what the Lancet report understands by actors willing to make change in the health care system [3].

The book could be classed more as a memoir than a textbook. The title is undeniably catchy, but what is described within comprises much more, so that the title does not quite do justice to the content. This fact elucidates how in our system, the education focus across all healthcare professions is on the inpatient sector, whereas in reality, the lion's share of patient care takes place in the outpatient sector.

In addition to those interested in the author team's areas of specialism and patients, this book is particularly recommended for three groups: medical and commercial management, HR, and senior physicians in clinics and other health care facilities. They will be given an idea of how and where they can "pick up" future health care professionals and what is needed to get into the system and, above all, stay in the system. The same applies to people who are active in health and education policy. Teachers at medical faculties can use these contributions to develop ideas on how to embed topics about health care provision and professional self-awareness into the curriculum, and how to be able to respond to them continually within the ever-increasing complexity of that curriculum.

In closing, it is fitting to let the author team speak for themselves, with words from their eleventh thesis: "Health is a social duty. How we go about this is part of the question of how we want to live" [2].

## Competing interests

The author declares that she has no competing interests.
